# Enhancing Molecular Network‐Based Cancer Driver Gene Prediction Using Machine Learning Approaches: Current Challenges and Opportunities

**DOI:** 10.1111/jcmm.70351

**Published:** 2025-01-13

**Authors:** Hao Zhang, Chaohuan Lin, Ying'ao Chen, Xianrui Shen, Ruizhe Wang, Yiqi Chen, Jie Lyu

**Affiliations:** ^1^ Postgraduate Training Base Alliance of Wenzhou Medical University Wenzhou Zhejiang China; ^2^ Wenzhou Key Laboratory of Biophysics, Wenzhou Institute University of Chinese Academy of Sciences Wenzhou Zhejiang China; ^3^ Wenzhou Longwan High School Wenzhou Zhejiang China

**Keywords:** cancer driver gene, deep learning, graph neural network, machine learning, protein–protein interaction, random walk

## Abstract

Cancer is a complex disease driven by mutations in the genes that play critical roles in cellular processes. The identification of cancer driver genes is crucial for understanding tumorigenesis, developing targeted therapies and identifying rational drug targets. Experimental identification and validation of cancer driver genes are time‐consuming and costly. Studies have demonstrated that interactions among genes are associated with similar phenotypes. Therefore, identifying cancer driver genes using molecular network‐based approaches is necessary. Molecular network‐based random walk‐based approaches, which integrate mutation data with protein–protein interaction networks, have been widely employed in predicting cancer driver genes and demonstrated robust predictive potential. However, recent advancements in deep learning, particularly graph‐based models, have provided novel opportunities for enhancing the prediction of cancer driver genes. This review aimed to comprehensively explore how machine learning methodologies, particularly network propagation, graph neural networks, autoencoders, graph embeddings, and attention mechanisms, improve the scalability and interpretability of molecular network‐based cancer gene prediction.

## 1 Introduction

Recent medical research has revealed that cancer results from the dysfunction of related dynamic systems [1]. From a systems biology perspective, cancer is triggered by state transitions in key cancer driver genes. These transitions disrupt molecular networks, such as gene regulatory networks, protein–protein interaction (PPI) networks and signal transduction pathways, which control molecular functions and cellular processes [2]. Additionally, the states of biomolecules, such as gene expression levels, exhibit complex dynamic behaviours that vary over time and in response to environmental conditions in cancer development [3].

**FIGURE 1:**
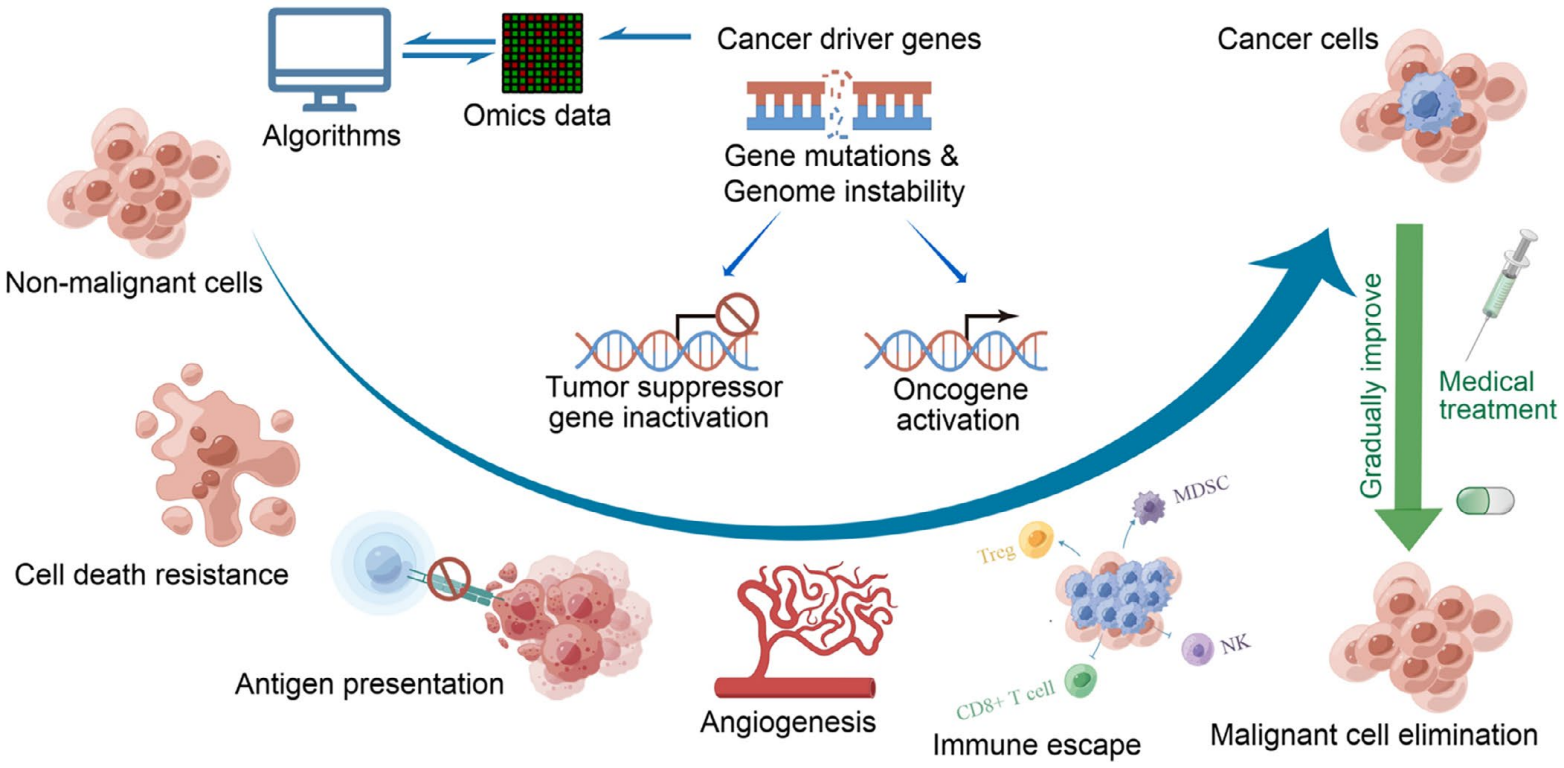
Cancer driver genes in tumour initiation and progression. Gene mutations or genome instability inducing tumour suppressor gene inactivation and oncogene activation can induce healthy cells to cancer cells. The tumours can grow and migrate through several mechanisms including cell death resistance, antigen presentation failure, angiogenesis, immune escape, gradually forming a tumour immune microenvironment. The reversal of cancer cell states through cancer gene targeting medicines is feasible and the study of which is still ongoing, highlighting the importance of cancer driver gene studies. The prediction of cancer driver genes based on omics data is therefore vital for cancer treatment. This figure is generated by the Figdraw tool provided by the HOME for Researchers website.

Cancer driver genes are those whose mutations confer a selective growth advantage or disadvantage to cells, promoting cancer progression or suppression (Figure 1) [4]. Oxidative stress is a driving force for DNA mutation, inducing DNA damage, genomic instability and increased cell proliferation (Figure 1) [5]. Immunotherapy is one of the most promising approaches in cancer treatment [6]. However, the experimental identification of cancer‐related genes for clinical applications is time‐consuming and costly. Owing to the controversies regarding the completeness of the catalogue of known cancer genes, the prediction of novel cancer genes remains critical in cancer genomics research [7]. Large‐scale cancer sequencing projects have produced extensive genomic and other molecular profiling data across various cancer types, facilitating the identification of new cancer genes. Many cancer driver gene databases and tools have been developed to promote research and clinical applications in oncology [8, 9, 10]. These resources are crucial in identifying and characterising mutations in cancer driver genes, which are essential for understanding the molecular mechanisms underlying cancer progression and for developing targeted therapies (Figure 1). For example, the Cancer Driver Drug Interaction Explorer integrates multiple gene–gene and drug–gene interaction databases, providing a comprehensive platform for identifying potential drug targets and repurposing candidates based on the specific mutations in different cancer types [11]. With the rapid advancement in the high‐throughput profiling of biomolecules, the development of molecular network biology has provided insights into exploring medicine intervention strategies with synergistic drug effects for cancer treatment [12]. Research has demonstrated that biologically relevant genes are more likely to influence a specific disease or a group of related diseases, and genes associated with similar phenotypes possibly interact with each other. Therefore, straightforward methods for prioritising cancer driver genes are molecular‐network‐based approaches. Studies have shown that targeting cancer driver genes, usually the crucial candidates for drug development, offer essential insights into drug discovery and repurposing [13].

Recent advancements in bioinformatics have prompted the development of various tools that facilitate studies, such as multi‐omics‐based, cancer driver gene and mutation and immune infiltration analyses [14]. Molecular network‐based approaches have emerged as effective tools in this domain, leveraging the complex interactions within biological systems to identify critical genes in cancer development [15]. These approaches have revealed that cancer is not merely a collection of individually mutated genes but a disease of disrupted molecular networks. Analysing the topology and dynamics of gene interaction networks aids in identifying the genes whose alterations significantly affect cellular processes and drive cancer progression. These methods consider the position and connectivity of genes within a molecular network to predict their likelihood to be cancer drivers. For example, genes with high centrality measures, such as degree or betweenness, are often considered potential cancer drivers owing to their significant influence on network dynamics [16]. Network‐based algorithms using PPI networks may have the potential to outperform frequency‐ and function‐based algorithms [8].

**FIGURE 2:**
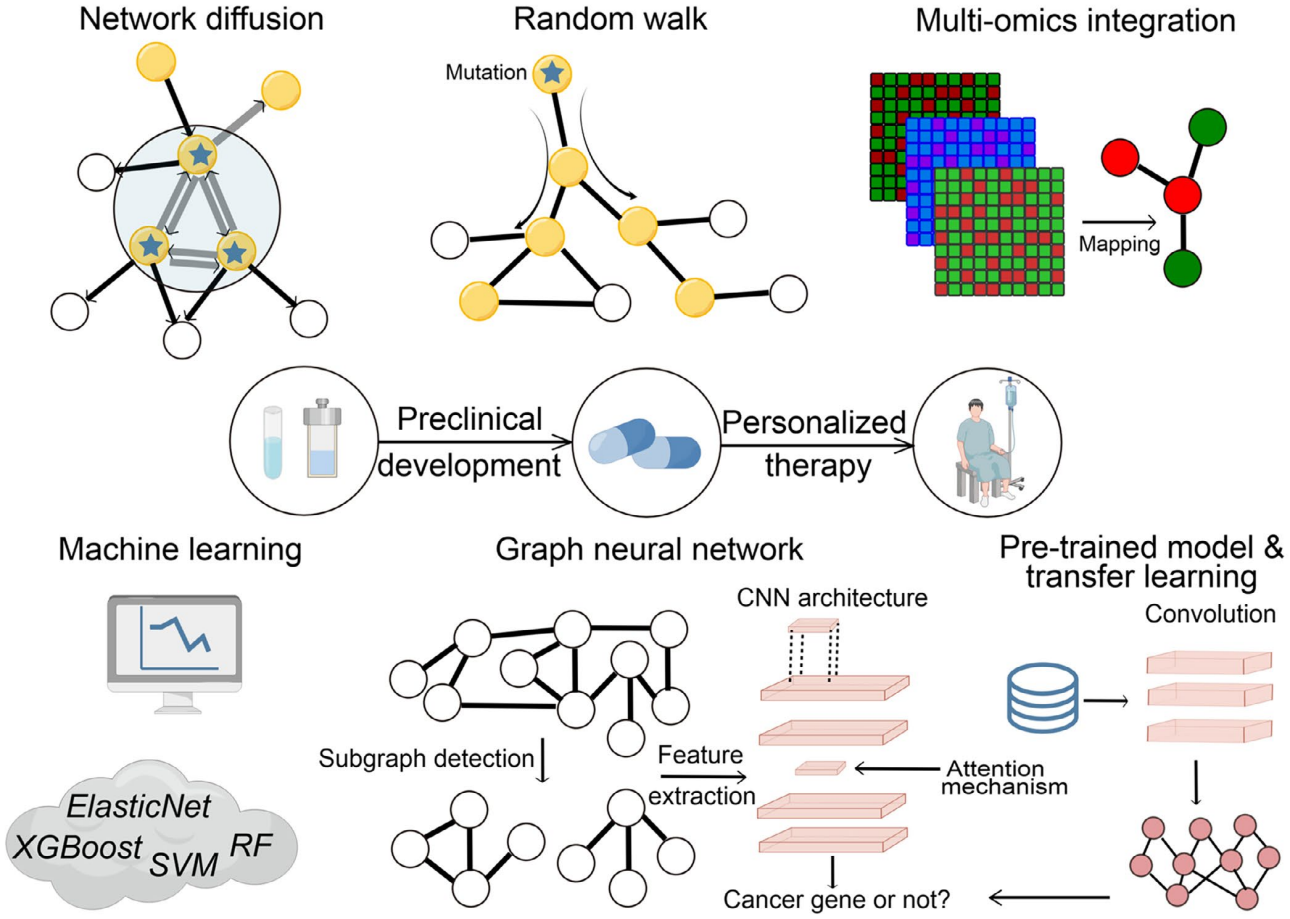
Overview of the primary approaches that can predict cancer driver genes. This figure is generated by the Figdraw tool provided by the HOME for Researchers website.

The recent advancements in this field have been marked by the integration of multi‐omics datasets and the application of machine learning algorithms to enhance prediction accuracy [10]. With the growing availability of high‐dimensional omics data and advances in machine learning, convolutional neural network and graph neural network (GNN) models have emerged as promising tools for improving the prediction of cancer driver genes. These deep models provide a novel strategy to dynamically learn representations of biological networks and efficiently integrate multi‐omics data, overcoming the limitations of classical machine learning approaches. This review discusses how the incorporation of network information into machine learning techniques, particularly deep learning methods, facilitates the prediction of cancer driver genes (Figure 2). The illustrations of the primary prediction strategies are presented in Table 1, along with the rational and primary applications or algorithms. Our work, therefore, helps researchers develop and use more efficient cancer driver gene prediction tools, which will ultimately contribute to the understanding of cancer genomics and advancing personalised medicine.

**TABLE 1:** Strategy of molecular network‐based cancer driver gene identification and the main applications.

Algorithm type	Essential input data type(s)	Rationale	Example applications
Heat diffusion	Protein–protein interaction (PPI) network and mutation	Mutations in cancer driver genes tend to have widespread effects across molecular interaction networks.	HotNet [17], HotNet2 [18]
Random walk	PPI network, mutation	Traversing molecular networks to prioritise genes that are closely related to known driver genes or mutation sources.	NBS [19], VarWalker [20], DawnRank [21], Subdyquency [22], Hierarchical HotNet [23], Driver_IRW [24]
Multi‐omics integration	Omics tabular data	The complexity of cancer arises from alterations across multiple molecular layers (e.g., genomics, transcriptomics, proteomics, epigenomics).	DriverNet [25], EntroRank [26]
Machine learning	Omics tabular data	The hidden patterns within the complex and high‐dimensional cancer data are difficult to analyse through statistical methods.	20/20+ [27], BetweenNet [28], LOTUS [29]
Deep graph embedding	Omics data and gene or protein interaction network	High‐dimensional interaction networks are transformed into lower‐dimensional representations while preserving the essential structural and functional relationships between genes.	RLAG [30], MNGCL [31], GM‐GCN [32], NMTF [33], ICDM‐GEHC [34]
Graph convolutional network	Omics data and gene or protein interaction network	The topology of graphs, including the direct and indirect relationships between nodes (genes), can be learned.	EMOGI [35], MTGCN [36], DGMP [37], MNGCL [31], NIGCNDriver [38], CGMega [39]
Autoencoders	Omics data and gene or protein interaction network	Cancer driver genes have hidden structures or patterns in multi‐omics data.	CancerGATE [40], SSCI [41]
Attention mechanisms	Omics data and gene or protein interaction network	Not all genes or interactions contribute equally to cancer. The attention allows to learn which genes or interactions are most relevant to the problem at hand.	CancerGATE [40], MRNGCN [42], HGDC [43], MODIG [44], CGMega [39]
Pretrained models or transfer learning	Omics data	Leveraging existing knowledge from other related tasks or datasets to improve the detection of cancer driver genes.	Not available

## 2 Network Propagation Algorithms

### 2.1 Heat Diffusion Algorithms

The network‐based propagation algorithm is efficient in disease gene prediction [45]. Network propagation amplifies biological signals based on the principle that genes associated with similar phenotypes possibly interact [46]. Ruffalo, Koyuturk and Sharan [47] have demonstrated that network propagation aids in predicting cancer driver genes, which form clusters within the network. Various network propagation algorithms, such as random walk, PageRank and heat diffusion, have been developed and successfully applied to address different biological challenges. The first heat diffusion‐based algorithm developed for predicting cancer driver genes was HotNet, which is based on the heat diffusion algorithm [17]. The efficiency of heat diffusion‐based methods is challenged by highly mutated or hub genes in molecular networks, such as *TP53* and *KRAS* (hotspots), which spread heat to neighbouring genes that may not be mutated or biologically relevant. This limitation has been addressed by HotNet2, which uses an insulated heat diffusion process, considering the direction of heat flow to improve the identification of biologically meaningful subnetworks [18]. HotNet2 captures the local topology around a gene, reducing false positives (e.g., star subnetworks dominated by highly mutated genes, such as *TP53* and *KRAS*) while detecting more subtle combinations of mutated genes. HotNet2 identifies rarely mutated genes by leveraging their interactions with frequently mutated genes. The vital advantage of HotNet2 is its ability to combine mutation scores with protein interaction networks to identify meaningful gene subnetworks, including those involving rare mutations, which are critical for understanding cancer biology.

### 2.2 Random Walk Algorithms

A random walk on a graph is a stochastic process where a ‘walker’ moves from one node (gene) to neighbouring node(s), selecting the next node at random based on certain transition probabilities. In cancer gene prediction, this process helps to identify nodes (genes) that are highly connected or frequently visited, implying their potential role as cancer driver genes. A common random walk method in biological network analysis is Random Walk with Restart (RWR). This variation includes the concept of ‘restarting’ the walker at a set of known driver genes, which allows the algorithm to consistently explore the network in proximity to these known genes while enabling specific exploration of the entire network. Random walk methods naturally integrate the topological features of PPI or gene regulatory networks with mutation data. The walker movement considers the direct interactions (local connections) and distant relationships (global network structure) of the genes. By restarting at known driver genes, RWR emphasises genes that are topologically related to known driver genes. This enables the identification of genes with less high mutation frequencies but strong connections to known driver genes. Rather than focusing on individual mutations, the random walk considers a broader network context, providing more robust predictions. Propagation‐based cancer research has rapidly improved the understanding of the association of gene sequence variations with gene expression changes through multiple diffusion processes [47, 48]. The representative tools include Network‐based Stratification [19], VarWalker [20], DawnRank [21], Subdyquency [22], Hierarchical HotNet [23] and UMG [49].

Specific recent methods (e.g., Driver_IRW [24]) improve the random walk by assigning weights to edges based on biological relevance (e.g., interaction strength and expression correlation) rather than treating all interactions equally, enabling more biologically meaningful predictions. They also ascribe varying transition probabilities to different edges in a cancer‐related network based on the degree of neighbouring nodes, which theoretically provides better performance than does non‐weighted network propagation.

## 3 Molecular Network‐Based Data Integration Algorithms

### 3.1 Network‐Based Heterogeneous Integration Methods

Although random walk approaches are efficient, they have certain limitations. The RWR algorithm favours genes with established characterisation or pronounced connection in the network, potentially overlooking less studied but significant cancer driver genes. In addition, random walk methods are usually limited to a single network type (e.g., PPI), which may miss crucial regulatory interactions that are not captured in that network. The basic random walk model can be adapted for more effective cancer driver gene prediction, for example, in heterogeneous networks [24, 50, 51, 52]. Random walk can be applied to heterogeneous networks that integrate different interaction forms, such as PPI, gene expression and epigenetic modifications. This enables a more comprehensive exploration of potential cancer driver genes. Random walk models can be adapted to integrate data from multiple sources (e.g., genome‐wide association studies, transcriptomics, epigenomics, pathways, gene–gene interactions, PPIs and pathway knowledge) to predict cancer driver genes by considering the different regulation aspects that influence gene function. Despite some limitations, random walk models, when combined with multi‐omics data and improved network quality, provide valuable insights into the genetic underpinnings of cancer [26, 53, 54, 55]. These methods use distinct strategies to identify cancer driver genes, offering valuable insights into the molecular mechanisms of cancer.

In addition, disease–gene networks are used in identifying disease genes, including cancer driver genes [56, 57]. Although related data resources are available, such networks are limited by the incompleteness of available datasets [58, 59]. Developing integrative frameworks based on molecular networks by collecting more multi‐omics data on cancer (e.g., somatic mutations, transcriptomics and epigenomics) would greatly improve the prediction of cancer driver genes.

### 3.2 Personalised Integration Methods

Cancer is a highly complex and heterogeneous disease, where the affected individuals may have distinct driver genes and experience varying outcomes, even when receiving the same treatment [60]. Population‐based approaches have limitations in translational medicine, as they usually fail to detect rare driver genes in small cohorts or individual patients. Consequently, exploring personalised cancer driver genes unique to each patient is crucial. In recent years, many researchers have proposed network‐based algorithms for identifying patient‐specific cancer driver genes for personalised diagnosis and treatment to overcome tumour heterogeneity at the population level [28, 31, 61, 62, 63, 64, 65, 66, 67, 68, 69, 70]. However, challenges persist in identifying driver genes for individual patients. Some network‐based methods may fail to make accurate predictions for certain patients. Interpreting trained deep learning models is challenging; however, their performance is better than that of machine learning models when applied to personalised omics data. Balancing accuracy and interpretability in cancer driver gene predictive models is crucial to improving patient outcomes. Model explainability techniques, such as Shapley Additive Explanations (SHAPs), provide this balance when coupled with deep learning models. In addition, attention mechanisms and explainable deep learning techniques significantly increase the mode interpretability while maintaining excellent prediction performance. This provides highly safe and effective clinical applications that can guide personalised treatments while being sufficiently transparent, ensuring trust among healthcare providers and patients. Further discussions on personalised cancer driver gene prediction are in other reviews [71, 72].

### 3.3 Common Concerns and the Current Status of Network Diffusion Approaches

Traditional molecular network‐based algorithms, particularly RWR‐based methods, offer effective tools for exploiting the topology of molecular interaction networks and prioritising novel cancer driver genes based on their connectivity with known cancer driver genes. However, there are drawbacks associated with network‐based approaches, such as the complexity and variability of biological networks, incomplete or low‐quality network and omics data and the low quality and incompleteness of available omics data. Furthermore, the computational intensities of certain network‐based methods may hinder their widespread adoption, particularly in settings with limited resources. In addition, the use of well‐known cancer driver genes may cause result biases, potentially affecting the predictive performance of certain genes. Moreover, such tools may suggest an excessive or insufficient number of candidate cancer driver genes or prioritise connected subnetworks, potentially overlooking isolated or understudied genes. The dilution of the distinguishing features of a driver gene by the dissimilar features of neighbouring genes during message aggregation is another challenge, reducing the effectiveness of standard GNNs in identifying cancer driver genes. Notably, the homophily assumption that nodes connected in a graph belong to the same class or share similar features may not hold for PPI or gene regulatory networks [73]. Similarly, the number of cancer driver genes is smaller than that of other genes in the molecular network, making them more likely to interact with non‐driver genes rather than only with other driver genes.

The current status of molecular network‐based approaches for predicting cancer driver genes is promising; however, it is in the evolving stage. The heterophilic nature of biomolecular networks introduces additional difficulties in the RWR approaches, underscoring the need for GNN‐based methods to handle heterophilic networks.

## 4 Artificial Intelligence Enhances Cancer Driver Gene Prediction Accuracy by Mining Deeper Information From Molecular Networks

Deep learning models are common in biomedical studies, such as molecular interactions [74, 75], toxicity [76], drug sensitivity [77], carcinogenicity [78] and medical diagnosis [79, 80]. The following section focuses on the application of deep learning models in cancer driver prediction based on molecular networks. Previously discussed traditional unsupervised and supervised machine learning‐based methods are not extensively assessed here [9, 81].

Random‐walk‐based models may be less efficient in identifying cancer driver genes. In contrast, the molecular network‐based prediction of cancer driver genes can be theoretically enhanced by leveraging deep learning and graph‐based models such as GNNs, autoencoders and graph embeddings. GNNs capture local and global gene interactions in PPI networks, improving the detection of cancer driver genes. Autoencoders reduce the dimensionality of high‐dimensional mutation data, enabling more efficient analysis. Graph embeddings simplify the network structure while preserving the essential relationships between genes. Additionally, attention mechanisms, such as Graph Attention Networks (GATs), allow models to prioritise important interactions and mutations, improving precision and interpretability. Integrating multi‐omics data through these models enhances prediction accuracy by harnessing an improved comprehensive view of cancer biology, making these methods effective tools for cancer driver gene prediction.

### 4.1 Machine Learning Methods That Use Network‐Related Metrics

PPI network‐derived metrics, such as degree, betweenness and closeness centrality, were used to compare the topological characteristics of cancer driver and non‐cancer genes [16]. Our findings revealed that known and predicted cancer driver genes by DORGE had significantly higher degrees, betweenness and closeness centrality than did non‐cancer genes, indicating that PPI network‐derived topological metrics are effective in predicting cancer driver genes [16]. Numerous approaches and tools, including 20/20+ and BetweenNet, have leveraged this information to predict cancer driver genes [27, 28]. The Learning Oncogenes and Tumour Suppressors model, for example, combines mutation frequency, functional impact and PPI network metrics to make such predictions [29]. However, a potential concern with models based on PPI network metrics is the risk of study bias. Cancer‐related genes are usually more focused on resulting in higher expression degrees within PPI networks.

### 4.2 GNNs

**FIGURE 3:**
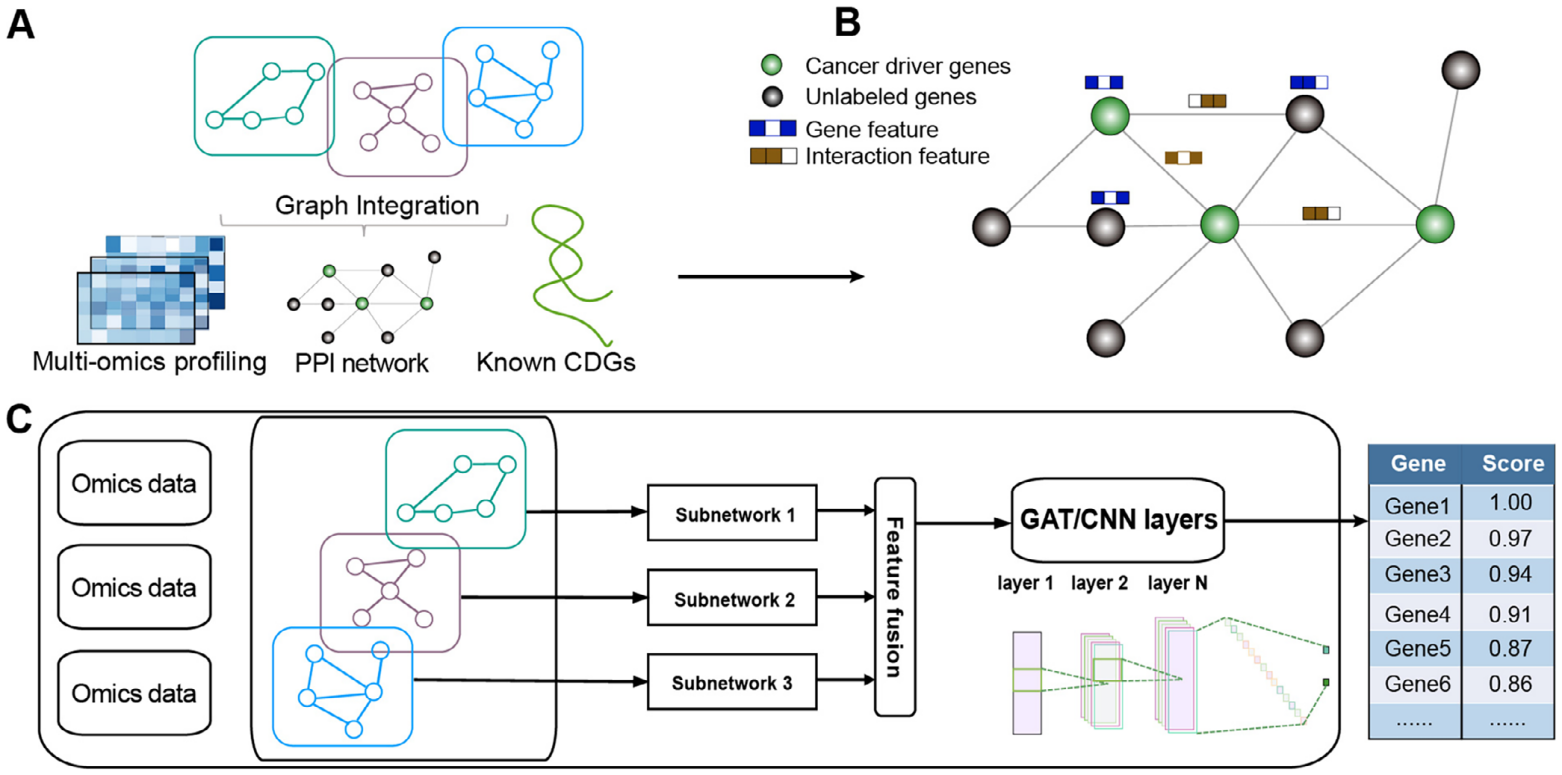
Schematic of network‐based graph embedding neural network framework. (A) Data preparation and integration. Gene interaction networks from multiple sources are merged into a single molecular graph, which is subsequently supplemented with transcriptome (or other omics) data along with information on known cancer driver genes. (B) Graph embedding for training. In this graph, nodes represent genes, edges represent gene interactions, node features correspond to multidimensional gene expression vectors and edge features encode gene interaction data from different sources as *n*‐dimensional binary vectors (*n* = 3, three sources in this plot). (C) Graph embedding‐based neural network framework for omics data integration. This framework is designed to enhance node embedding extraction within a graph for node classification tasks. The framework processes molecular interaction graphs and is trained on these graphs. The node embeddings from all subgraphs are used to generate features separately that can be coupled with a convolutional neural network or graph attention network model to generate the final gene‐ranked list, representing their likelihood (equivalent to ranking score) of being associated with cancer. CDGs, cancer driver genes.

GNNs, designed to operate directly on graph structures, are particularly suitable for modelling PPIs owing to the graph structure of interaction networks (Figure 3A). The pioneering GNN model EMOGI incorporates multi‐omics features of cancer genes within PPI networks to predict novel cancer genes [35]. EMOGI represents each gene as a vector summarising multi‐omics data across various cancer types from The Cancer Genome Atlas (TCGA) datasets. Subsequently, it models gene–gene interactions from predefined generic PPI networks using a graph convolutional network (GCN). However, EMOGI does not consider the relevance of cancer physiology in the predefined graph topology and connectivity patterns. The MTGCN method alleviates the challenge by constructing a multichannel GCN network that combines driver gene identification with link prediction [36]. In addition, GNNs are used by many other available algorithms, including those coupled with contrastive learning (e.g., MNGCL [31]), the Multilayer Perceptron model (e.g., DGMP [37]), as well as multi‐omics integration (Song et al. [82]). Furthermore, personalised approaches, such as NIGCNDriver [38], utilise graph convolution networks to predict cancer driver genes personalised to individual patients. In addition to PPI networks, GNNs utilise miRNA–gene networks to predict cancer driver genes [32]. These studies illustrate GNNs' capacity to integrate complex biological data in gene interaction networks to prioritise crucial driver genes. Nevertheless, the available network‐based approaches rely on unidimensional data from single omics sources or force the integration of genetic features and network topology into a single feature type, potentially leading to a loss of valuable information. To address this, Li et al. [39] recently developed CGMega to construct a multi‐omics representation graph, where genes are represented as nodes and edges are defined by PPIs between genes. By integrating PPI networks, CGMega reveals critical cancer gene modules involved in cancer progression [39].

GNNs use a fixed weighting scheme for aggregating features from neighbouring nodes, and all neighbours are treated equally during feature aggregation. This can be suboptimal in complex biological networks where some interactions may be more significant than others for predicting cancer driver genes. Graph attention autoencoders (GAEs) may be more effective tools for weighted graph‐based learning or complex biological networks, as discussed below.

### 4.3 Deep Graph Embedding

Graph embeddings enable a convenient and efficient representation of interactions in the weighted PPI network. For example, deep graph embedding techniques allow for gene mapping into an embedding space where similar genes are closer. Graph embeddings enable the integration of various types of biological data (such as gene expression, mutations and protein interactions), leading to an improved informative feature representation of cancer driver genes. Deep graph embedding methods can be enhanced through node embedding strategies (Figure 3B). These embeddings capture local and global network structures, which are subsequently used for predicting cancer driver genes based on conventional convolutional neural network or GAT infrastructure (Figure 3C). Node2Vec, a popular algorithm for graph‐based embedding, is commonly used in cancer driver gene predictions owing to its ability to learn low‐dimensional representations of nodes (genes) in large biological networks [30, 31, 32, 33, 34, 83, 84]. For example, through Node2Vec, Zong et al. [83] predicted cancers using genetic and clinical data in a network‐based framework, enabling them to encode gene relationships into feature vectors and improving the performance of the prediction models for cancer types. The integration of genetic and clinical data using Node2Vec increases the model's predictive accuracy. Cancer driver genes that have greater contribution based on SHAP values (key features in cancer prediction) can be the prioritised candidates.

Node2Vec, a classical network‐embedding method, may not accurately simulate long‐distance nodes with structural similarities because of its limited sampling length during random‐walk iterations. To address this limitation, Chu et al. [84] used the struc2vec model to vectorise newly constructed network nodes. The struct2vec model encodes structural similarities by creating multilayer graphs that generate structural contexts for the nodes. Compared with the performance of Node2Vec, struc2vec effectively identifies distant gene pairs that exhibit similar structures.

Approaches based on graph embedding usually outperform traditional machine learning models, particularly in complex biological data. The generated embeddings can correspond to biologically meaningful features that capture the functional properties of cancer driver genes, such as their roles in specific pathways, facilitating the identification of functionally relevant cancer driver genes. However, similar to other machine learning models, interpreting gene network embeddings is usually challenging. The embeddings may not provide clear insights into the basis for classifying certain genes as cancer driver genes, complicating the experimental validation of predictions.

### 4.4 Autoencoders

Autoencoders are unsupervised deep learning models for compressing high‐dimensional omics data (e.g., somatic mutation profiles) into lower dimensional representations to capture essential information. The latent space learned by autoencoders can be used as input features for downstream models such as GNNs or classification models. Autoencoders, particularly sparse and denoising variants, extract vital features from high‐dimensional noisy datasets, increasing the robustness of cancer driver gene predictions.

Autoencoders also incorporate molecular interaction networks. Network‐based autoencoders, which combine neural networks and graph structures, have been proven effective in identifying cancer driver genes [40, 41, 85]. By leveraging the potential of autoencoders to capture latent representations and the structure of biological networks, these models can enhance the prediction of the crucial driver genes involved in cancer progression.

For example, CancerGATE utilises graph attention autoencoders to predict cancer driver genes by incorporating gene interaction networks [40]. The method combines autoencoders' feature extraction capabilities with GNNs' ability to learn from structured data. Chang et al. [85] applied sparse autoencoders to analyse PPI networks, focusing on cancer‐related genes and potential drug targets. Sparse autoencoders are designed to enforce sparsity in their hidden layers, helping to filter noise and retain only the most significant features. This model prioritises genes by extracting essential information from high‐dimensional interaction data.

The advantages of autoencoders include enhanced prediction accuracy, feature extraction ability and noise reduction in a self‐supervised manner. By leveraging network structures and integrating multi‐omics data, the aforementioned models can improve the prediction of cancer driver genes compared with traditional methods that usually treat genes as independent variables. Autoencoders also have notable limitations. The integration of network‐based autoencoders and GNNs requires careful model design and parameter tuning. This complexity makes training and interpreting these models challenging. Although autoencoders improve prediction accuracy, the latent representations they learn may not always be easily interpretable, complicating the biological interpretation of the prediction results.

### 4.5 Attention Mechanisms

GNNs use a fixed‐weighting scheme to aggregate features from neighbouring nodes. This implies that all neighbours are treated equally during feature aggregation, which can be suboptimal in complex biological networks, where some interactions may be more significant than others for predicting cancer driver genes. In contrast to GAEs, which leverage attention mechanisms to dynamically assign significance to different nodes based on their relevance, traditional GNNs lack this flexibility [86, 87], possibly causing the inclusion of noisy or irrelevant information in the feature representation and potentially diluting the signal to identify driver genes. GNNs are less effective in integrating various types of biological data (e.g., somatic mutations, transcriptomics and gene interactions) within a single framework. This integration is simple in GAEs, which leverage attention mechanisms to merge information from multiple data sources more effectively. The fixed aggregation scheme in GNNs causes limited interpretability regarding the features or interactions that drive the predictions. However, GAEs, with their attention weights, provide clearer insights into the nodes and edges that influence the model output, which is crucial for understanding cancer biology [88].

Attention mechanisms empower models to focus on the most significant parts of the large‐scale input data, improving the understanding of how certain genes contribute to tumorigenesis by focusing on their relative significance within the biological network [89]. In graph networks, attention is used to prioritise interactions between specific genes based on their significance in cancer progression. GATs incorporate attention into GCNs, allowing the model to weigh the significance of different edges (gene interactions) when predicting cancer driver genes. Genes with more impactful interactions are of greater significance in the final driver gene prediction. Cancer driver genes affect various biological pathways by interacting with other entities within the molecular networks. In cancers, functional interactions between cancer driver genes, such as PPIs and gene expression patterns, can be rewired. Several recent studies have demonstrated the significance of attention mechanisms in predicting cancer driver genes coupled with autoencoder architecture (CancerGATE [40]), graph convolutional networks (EMGNN [90], MRNGCN [42] and HGDC [43]), multi‐omics integration (MODIG [44]) and transformer architecture (CGMega [39]). Attention mechanisms provide interpretable insights into the genes or interactions that are most critical for prediction, making model decisions more transparent and interpretable. This is valuable for researchers and clinicians who require an understanding of predictive logic.

### 4.6 Transfer Learning and Pretrained Models

Transfer learning can be leveraged by pretraining models on large, publicly available biological datasets (e.g., TCGA or GTEx) and fine‐tuning the model on cancer‐specific data [91]. Pretrained models can be developed using large‐scale multi‐omics datasets, such as transcriptomics, genomics, proteomics and interactome data [92, 93, 94]. The models are trained to learn patterns from large, diverse biological datasets. For cancer driver gene prediction tasks, models should be fine‐tuned on cancer‐specific datasets, enabling them to transfer their learned knowledge to predict potential cancer driver genes in new smaller datasets with better accuracy. Potential pretrained models for cancer driver gene prediction may use graph‐based techniques such as GCNs. These models can be trained on vast gene interaction networks to capture the intricate relationships between genes and their functions. Subsequently, the pretrained GCNs are fine‐tuned using cancer‐specific data to identify genes that are central to cancer pathways and have mutation patterns indicative of driver genes. While pretrained models offer significant potential for future tasks in cancer driver gene prediction, to the best of our knowledge, there are no known applications of molecular network‐based transfer learning studies. However, we expect the availability of such research.

### 4.7 Concerns About Model Performance Comparison

Different models can generate ranked gene lists for further analysis and evaluation. However, only a few studies have evaluated the performance of different methods within unified evaluation metrics and data inputs [16]. Although we systematically reviewed the current status of the available network‐based cancer driver gene prediction tools, this review does not consider the performance comparison between them owing to the extensive work.

Accuracy, the area under the receiver operating characteristic curve, recall (or sensitivity) and precision are the most frequently used metrics. For class‐imbalanced data, the precision, recall, F1‐score and area under the precision‐recall curve are more appropriate. Genuine gold‐standard cancer driver gene lists are still unavailable. Commonly used cancer gene sets, such as the Cancer Gene Census, which is a manually curated gene set, can be used as true‐positive gene sets. Similarly, defining a negative non‐cancer gene set is essential. However, there are no commonly used non‐cancer driver gene lists. Previously published non‐cancer driver genes may be a candidate gene set [16]. Considering that known evaluation research does not include most of the tools mentioned in this article [8], a unified performance evaluation for these tools and the tools evaluated in the previous study is useful. Thus, this task requires substantial effort, which can be performed separately in future studies.

## 5 Challenges and Perspectives

The identification and interpretation of cancer driver genes is a critical challenge in cancer biology. Large‐scale cancer sequencing initiatives, such as TCGA, have facilitated the development of numerous computational methods to address this concern. Despite substantial advancements, the application of machine‐ and deep learning models may enhance cancer driver gene prediction accuracy greatly based on molecular networks. Emerging technologies, such as liquid biopsy, which aids in detecting tumour DNA (ctDNA) in blood samples, reveal cancer‐driving mutations even in the early stages of cancer when the tumour is excessively small to be detected through imaging. The data generated from such technologies are particularly suitable for personalised and deep learning‐based cancer driver gene prediction approaches, which is relevant for early cancer detection, risk evaluation, targeted therapy and prognostic evaluation. Cancer driver gene prediction enables the development of targeted therapies and also informs the development of novel effective drugs. Thus, an excellent cancer driver gene prediction system could significantly enhance the effective management of cancer. This review summarises the various network‐based methods for identifying new cancer driver genes. However, several challenges persist, which should be considered in future studies.

Despite the advancements of network‐based methods, they have certain limitations. A primary challenge in training network‐based machine‐ and deep learning models for cancer driver gene prediction is the limited availability of large‐scale paired multi‐omics datasets. Omics datasets, including genomic, transcriptomic, proteomic and epigenomic data, are essential for training models to learn comprehensive biological relationships. The scarcity of such data hampers the model's ability to generalise in different cancers and make accurate predictions. In addition, biomedical datasets are usually collected using various experimental techniques and preprocessing methods. This inconsistency complicates the integration of multi‐omics datasets using network‐based models. Consequently, the models face challenges in learning from diverse modalities and deriving meaningful insights across datasets. In addition, molecular networks, including PPI and gene regulatory networks, are inherently sparse and high dimensional. Training deep learning models on such data require substantial computational power, and the data sparsity may cause overfitting, reducing the model performance. Graph models alleviate these limitations because they focus only on local data structures. For example, a random walk propagates information to neighbours, whereas GNNs learn node representations from a node's local neighbourhood rather than relying on a full adjacency matrix. Similarly, predictions based on deep learning vary depending on the PPI network used for training. Training deep learning models on large biological networks is computationally expensive. The iterative nature of learning from graph‐structured data, particularly in GNNs, increases the time and resources for model development, hindering the practical application of these models. Finally, most of the GNN and other approaches rely on undirected graphs derived from PPI networks. However, gene regulatory processes and signalling pathways are inherently directional, providing critical insights for identifying disease‐associated genes. Thus, developing methods that effectively utilise the directional information in digraphs is crucial to improving the precision of node classification tasks.

Network‐based machine learning models, particularly deep learning, usually act as ‘black boxes’, making it challenging to interpret how specific features, interactions or pathways impact the identification of cancer driver genes or how specific features or cancer driver genes impact the identification of cancer‐related pathways. This lack of transparency limits their adoption in clinical settings where interpretability is critical for validating predictions and making informed decisions. Recent efforts to make deep learning models more interpretable offer a promising direction for network‐based cancer driver gene predictions. Techniques such as attention mechanisms and explainable deep learning methods help identify the interactions or network pathways that are most relevant to the model's predictions and are preferred in future studies. In addition to interpreting the significance of individual molecular features in model outcomes, identifying the regulatory roles across multiple omics features using self‐supervised learning is vital. Recognising the regulatory associations between molecular features at different scales may help reveal more intricate mechanisms underlying tumorigenesis and cancer progression. Improved interpretability facilitates model validation and increases confidence in its clinical applicability.

With the advance in multi‐omics profiling technologies, more comprehensive datasets that capture various biological layers (e.g., genomics, transcriptomics, proteomics and epigenomics) are becoming available and could be valuable for network‐based deep learning models. These datasets will enable better training of network‐based deep learning models, allowing them to integrate diverse data types and capture the intricate relationships between molecular features (e.g., somatic mutations) and cancer progression. Although binary machine learning models are common tools for predicting cancer drivers, they require large, unbiased training datasets. Owing to the lack of a reliable dataset of non‐cancer driver genes, the class imbalance limitation in machine learning tasks remains evident, which may affect the accuracy of model training and validation. One‐class or unsupervised models may also facilitate such task [95], as they do not need to curate positive and negative examples for training. This strategy also helps address the class imbalance challenge and the uncertainty regarding the definition of non‐driver genes. However, although the current understanding of well‐established cancer driver genes is limited, they can significantly enhance learning outcomes when leveraged appropriately.

In addition to modelling omics data, GNN and other deep learning models can be used to preprocess data and handle omics data fusion, which are usually challenging in traditional models. For example, integrating attention mechanisms into traditional cancer driver prediction tools such as MutSigCV could refine predictions, highlighting the mutations or genetic features that actually drive cancer. Furthermore, known prediction tools that already handle multi‐omics data can incorporate GNNs to model complex relationships between different omics layers, enabling predictive models to capture more intricate relationships that may be missed by traditional tools. Although GNN can be scaled to large and complex datasets, future tools incorporating attention mechanisms can help reduce the computational load by focusing only on the significant parts of the data during training, making the predictive models more efficient and faster for practical use.

We envision that transfer learning and pretrained models are among the most common techniques for enhancing the accuracy of cancer driver gene prediction. Leveraging models pretrained on increasingly available omics datasets and adapting them to smaller cancer‐specific datasets improve the performance of cancer‐type‐specific or personalised cancer driver gene predictions. Such models can be used to identify complex patterns in large genomic datasets that are challenging to detect using traditional methods. In addition, Explainable Artificial Intelligence, particularly SHAP and local interpretable model‐agnostic explanations, facilitates the interpretation of network‐based deep learning models, allowing clinicians to extract crucial cancer drivers and understand the rationale underlying a specific prediction task.

This review provides directions for future bioinformaticians, clinical practitioners and cancer biologists. Future research could perform performance comparisons between network‐based prediction and other tools in a unified data context, which is not covered in this review. In addition, other deep learning infrastructures, such as recurrent neural networks (RNNs), may also be used in future cancer driver gene prediction tasks. RNNs may be particularly useful for capturing long‐range dependencies, enabling the identification of effects that spread across multiple interactions within the network. Although the use of RNNs to predict cancer driver genes is currently unavailable, we anticipate broader applications in future research. Functional experiments, including cell proliferation‐ and animal‐based, can also validate the identified cancer driver genes in specific cancers, given that the roles of many predicted cancer driver genes in cancer development remain unclear. Moreover, cancer driver genes can be assessed based on their roles in selection during tumour evolution. Gaining insights into tumour evolution necessitates computational analysis of intratumour heterogeneity. The safe delivery of therapeutic compounds or gene products to tumour cells is highly crucial in clinical practice, as commonly used nanoparticles may harm human health via ferritinophagy [96, 97]. Future research should prioritise the selection of efficient delivery systems, such as exosomes [98] for cancer drivers, to address the concerns in clinical trials. Significant advancements have been made recently in single‐cell sequencing and other single‐cell‐level experimental techniques. These innovations are garnering significant attention in investigating cancer development and are expected to offer additional tools for identifying and studying cancer driver genes.

## 6 Conclusions

Network‐based deep learning models offer significant promise for improving the prediction of cancer driver genes; however, they are limited by data availability, network complexity and model interpretability. However, advances in multi‐omics integration and graph‐based deep learning techniques have provided insights into overcoming these concerns. By addressing these challenges, network‐based deep learning models can play a transformative role in understanding cancer genomics and advancing personalised medicine.

## Author Contributions

**Hao Zhang:** visualization (equal), writing – original draft (equal), writing – review and editing (equal). **Chaohuan Lin:** writing – review and editing (equal). **Ying'ao Chen:** visualization (equal). **Xianrui Shen:** resources (equal). **Ruizhe Wang:** resources (equal). **Yiqi Chen:** resources (equal). **Jie Lyu:** conceptualization (lead), funding acquisition (lead), supervision (lead), writing – original draft (equal), writing – review and editing (equal).

## Ethics Statement

The authors have nothing to report.

## Consent

The authors have nothing to report.

## Conflicts of Interest

The authors declare no conflicts of interest.

## Data Availability

The authors have nothing to report.
